# Biogenesis and Function of Circular RNAs in Health and in Disease

**DOI:** 10.3389/fphar.2019.00428

**Published:** 2019-04-26

**Authors:** George Haddad, Johan M. Lorenzen

**Affiliations:** Division of Nephrology, University Hospital Zürich, Zurich, Switzerland

**Keywords:** circular RNA, non-coding RNA, exosome, platelet, kidney

## Abstract

Circular RNAs (circRNAs) are a class of non-coding RNA that were previously thought to be insignificant byproducts of splicing errors. However, recent advances in RNA sequencing confirmed the presence of circRNAs in multiple cell lines and across different species suggesting a functional role of this RNA species. CircRNAs arise from back-splicing events resulting in a circular RNA that is stable, specific and conserved. They can be generated from exons, exon-introns, or introns. CircRNAs have multifaceted functions. They are likely part of the competing endogenous RNA class. They can regulate gene expression by sponging microRNAs, binding proteins or they can be translated into a protein themselves. CircRNAs have been associated with health and disease, some with disease protective effects, some with disease promoting functions. The widespread expression and disease regulatory mechanisms endow circRNAs to be used as functional biomarkers and therapeutic targets for a variety of different disorders. In this concise article we provide an overview of the association of circRNAs with various diseases including cancer, cardiovascular and kidney disease as well as cellular senescence. We conclude with an assessment of the current status and future outlook of this new field of research that carries immense potential with respect to diagnostic and therapeutic approaches of a variety of diseases.

## Introduction

Cellular ribonucleic acids are a family of coding and non-coding sequences. Merely 1–2% of the human genome is transcribed into RNA transcripts, which are translated into protein (Lorenzen and Thum, 2016; Brandenburger et al., 2018). Aside from mRNA which represents 3–7% (Hastie and Bishop, 1976; Carter et al., 2005) of the total RNA species in mammalian cells, the majority of the RNA species are of the non-coding category that include ribosomal RNA (rRNA) (80–90% Wolf and Schlessinger, 1977; Duncan and Hershey, 1983; Palazzo and Lee, 2015), transfer RNA (tRNA) (10–15%; Waldron and Lacroute, 1975; Palazzo and Lee, 2015), long non-coding RNA (0.06–0.2%; Mortazavi et al., 2008; Palazzo and Lee, 2015), microRNA (0.003–0.02%; Bissels et al., 2009; Palazzo and Lee, 2015), and circular RNA (circRNA) (0.002–0.03%; Salzman et al., 2012; Palazzo and Lee, 2015) among others. Non-coding RNAs (ncRNAs) are separated into long ncRNAs (lncRNAs, ≥200 nucleotides) and small ncRNAs (≤200 nucleotides). Small RNAs including microRNAs, which lead to the repression of gene/protein expression and/or translational inhibition of protein synthesis by post-transcriptional binding of the 3′-untranslated region (UTR) of mRNA targets, have been extensively studied over the past several years (Lorenzen and Thum, 2016; Brandenburger et al., 2018). In contrast, only little information is available regarding the functional importance of long non-coding RNAs (lncRNAs). It is becoming evident that lncRNAs are important epigenetic regulators of tissue homeostasis during development and disease (Lorenzen and Thum, 2016; Brandenburger et al., 2018). LncRNAs may be categorized as: (1) sense or (2) antisense, (3) bidirectional promoter (transcribed within 1 kb of promoters antisense to protein-coding transcript), (4) intronic, (5) intergenic, or (6) enhancer-associated (transcribed from an enhancer region of a protein-coding gene) (Lorenzen and Thum, 2016; Brandenburger et al., 2018). In addition, circular RNAs have been identified as part of the lncRNA class. These RNAs are characterized by a circular structure in which the 3′ and 5′ ends are covalently linked. In the present article we aim to elaborate on the biogenesis of this newly identified RNA class as well as discuss its potential function.

Circular RNAs are diverse RNA species that are found in all live forms from archaea to humans (Danan et al., 2012; Jeck et al., 2013; Haque and Harries, 2017). Although, circRNAs have been discovered over 20 years ago they were initially dismissed for having low abundance or resulting from splicing errors (Salzman et al., 2012; Jeck and Sharpless, 2014; Zheng et al., 2016). However, recent advancements in high throughput sequencing revealed the presence of circRNAs in mammalian cells, across various cell lines and many transcripts are abundant and stable (Jeck and Sharpless, 2014; Haque and Harries, 2017). CircRNAs are generated through a mechanism known as back-splicing “tail” to “head” whereby an exon at the 3′end of a gene is back-spliced to an exon at the 5′end of the gene resulting in a circular RNA form (Cocquerelle et al., 1993). CircRNAs are dispersed throughout the genome. They can arise mainly from exons but circRNAs deriving from inter- or intragenic, and intronic regions as well as antisense sequences have been reported (Cocquerelle et al., 1993; Lan et al., 2016).

It is imperative to distinguish valid circular RNA from artifact circular RNA. Jeck and Sharpless (2014) argued that backsplice events can occur not only from exonic circRNAs but also can be generated through other mechanisms such as RT-PCR template switching, RNA trans-splicing, as well as tandem duplication. Therefore, methods to validate the circularity of an RNA are essential.

Several methods have been described to separate linear from circRNAs. In general circRNAs do not have a 3′end as compared to linear exonic RNA and they migrate slower in a two-dimensional polyacrylamide gel (Tabak et al., 1988). RNA degradation by RNase H or weak hydrolysis can offer a more conclusive method for RNA circularity by linearizing the circRNAs into one product (Capel et al., 1993). In addition, other enzymes that degrade RNA can be employed such as RNase R exonuclease, and terminator exonuclease which have no effect on the circRNAs but degrade the majority of linear RNA (Suzuki et al., 2006; Hansen et al., 2011, 2013; Jeck and Sharpless, 2014).

**FIGURE 1:**
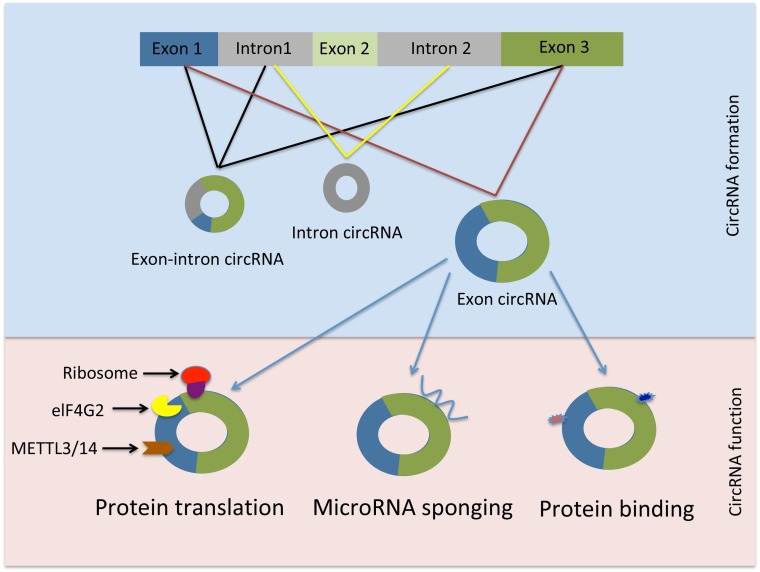
Schematic representation of circRNAs generation and function. elF4G2, initiation factor eukaryotic translation initiation factor 4 gamma; METTL3, methyltransferase-like 3; METTL14, methyltransferase-like 14.

Similarly, circRNAs must be distinguished from RNA lariats which are generated through canonical RNA splicing and resemble exonic circRNAs. However, lariat RNAs are distinguished by the presence of a 2′–5′junction in contrast to the 3′–5′linkage present in exonic circRNAs. Due to this linkage difference RNA lariats can be depleted by treatment with RNA lariat debranching enzyme followed by exonuclease digestion (Ruskin and Green, 1990; Zhang et al., 2013; Jeck and Sharpless, 2014). CircRNA biogenesis is schematically shown in Figure 1.

### Cellular Source of circRNA

Genome-wide analyses establish a strong evidence of circRNA presence in various cell types (Salzman et al., 2012; Zhang et al., 2013; Jeck and Sharpless, 2014). The expression of circRNAs is prevalent, specific, stable, and displays a temporal and spatial regulation suggesting tissue specific function under physiological and pathological conditions (Wang et al., 2014; Xu et al., 2017; Fang, 2018). They have higher expression levels in low-proliferating cells such as in the brain as compared to high-proliferating cells of the liver (Bachmayr-Heyda et al., 2015). Moreover, in hematopoietic cells including progenitors, differentiated myeloid and lymphoid cells, circRNA expression can be cell specific and increases during cellular maturation. Interestingly, enucleated cells such as red blood cells and platelets appear to express higher levels of circRNAs as compared to nucleated hematopoietic cells (Nicolet et al., 2018). It was found that platelets, in particular, express the highest number of circRNAs, almost twice as much as erythrocytes and 5 times more than granulocytes (Nicolet et al., 2018). Taken together, this suggests that circRNAs may play an important role in maintaining erythrocyte and platelet function in responding to micro-environmental cues and to cross-talk with other cell types through the packaging of circRNAs into microvesicles, or exosomes (Nicolet et al., 2018). Exosomes are vesicles that pinch off the plasma membrane, sized between 40 and 150 nm in diameter where intracellular materials can be packaged and delivered as messenger shuttles between cells (Properzi et al., 2013; Raposo and Stoorvogel, 2013). Interestingly, the expression levels of select circRNAs in exosomes derived from cancerous tissue is higher than in non-cancer derived exosomes (Li Y. et al., 2015). Exosomes are generated through the exosomal pathway that involves the expression of distinct protein markers not present in other vesicles of comparable size. These markers include tetraspanins (TSPAN27, 28, and 29), Tsg101 and Alix from the Rab family of proteins, and heat shock proteins such as HSP70 (Bobrie et al., 2011). The prevalence of circRNAs in extracellular exosomes is proposed to serve as reliable biomarkers in diagnosing various diseases and also assessing the efficacy of a certain treatment (Van Der Pol et al., 2012; Abu and Jamal, 2016).

## Exonic circRNA Hypothesized Functions

The evidence on the molecular and cellular functions of circRNAs is emerging. However, regarding the vast majority of circular RNA species their biogenesis, targets and regulation are still not well understood (Vicens and Westhof, 2014; Salzman, 2016). The cellular location of certain circRNAs may provide an indication of their function. Many exonic circRNAs with retained introns as well as circular intronic RNAs (ciRNAs) are found predominantly in the nucleus suggesting a transcription regulatory function (Li Z. et al., 2015; Huang et al., 2017), whereas, circRNAs that are generated from exons only are mostly found in the cytoplasm, which may insinuate a role in post-transcriptional gene regulation (Salzman et al., 2012; Jeck et al., 2013; Wilusz, 2018).

The circRNA-sponging function of miRNA is well characterized in the literature (more examples are provided in this review in the following sections). However, it should be noted that merely binding of circRNA to a miRNA does not always result in miRNA suppression. For example, when ciRS-7 is bound to miR-671 it becomes susceptible to cleavage by Argonaute 2 (AGO2), thus releasing miR-7 in the process. Therefore, circRNAs may act as a miRNA reservoir or mediate their transportation (Kristensen et al., 2018).

Interestingly, certain circRNAs have the potential to be translated into protein in a process that requires N^6^-methyladenosine (m^6^A) RNA base modification, the initiation factor eukaryotic translation initiation factor 4 gamma (elF4G2), methyltransferase-like 3 (METTL3), and methyltransferase-like 14 (METTL14). Mass spectrometry and computational prediction analyses demonstrated that there are hundreds of endogenous circRNAs with the potential to be translated into protein (Yang et al., 2017). For instance, circ-FBXW7 encodes a 21 kDa protein known as F-Box and WD Repeat Domain Containing 7 (FBXW7) – 185aa. Overexpression of this novel protein reduced cellular proliferation and repressed glioma tumorigenesis (Yang Y. et al., 2018).

Moreover, circRNA function may be extended to include protein-binding activity and it has been suggested that circRNAs are likely to be involved in protein binding, sorting, sequestering, and modulating protein-protein interactions through scaffolding properties. Based on bioinformatic analyses many circRNAs are predicted to harbor RNA binding protein sites (Du et al., 2017b). However, due to the tertiary structure of circRNAs the protein binding function appears to be a more complicated process than previously thought (Du et al., 2017b). Nevertheless, there are few reports demonstrating the protein binding capacity of circRNAs. For instance, Argonaute proteins, which are involved in miRNA trafficking, were found to strongly interact with human *circRNA cerebellar degeneration-related protein 1 transcript* (CDR1as) by using the technique photoactivatable-ribonucleoside-enhanced crosslinking and immunoprecipitation or PAR-CLIP (Memczak et al., 2013). Additionally, the circRNA circMbl generated from exon 2 of the splicing factor *muscleblind* (MBL/MBNL1) in both drosophila and humans carries highly conserved binding sites for the RNA binding protein MBL in both the flanking introns and within the circularized exon 2. The authors further demonstrated a strong and specific binding of MBL to the circRNA, and this interaction was essential for increased circMbl expression by exogenous expression of MBL (Ashwal-Fluss et al., 2014). CircRNAs as well as their various functions are summarized in Table 1.

**Table 1:** Summary of circRNAs and their functions in health and disease.

circRNA	Species	Function	Target microRNA	References
**Circrnas as Regulator of Mirna Function**				
CDR1as	Mouse Zebrafish	Impair brain development	miR-7	Memczak et al., 2013
Sry	Human	Testis-specific	miR-138	Hansen et al., 2013
circHIPK3	Human	Regulates cell growth	miR-124	Zheng et al., 2016
circCOL3A1-859267	Human	Collagen type expression	miR29c	Peng et al., 2018
circZfp609	Mouse	Suppresses the myogenic differentiation	miR-194-5p	Wang et al., 2019
circRNA.2837	Rat	Render neurons susceptible to neurological damage	miR-34 family	Zhou et al., 2018b
**Oncogenic Circrnas**				
has_circ_0078710	Human	Involved in hepatocellular carcinoma development by increasing expression of HDAC and CDK2	miR-31	Xie et al., 2018
circFBLIM1	Human	Hepatocellular carcinoma	miR-346	Bai et al., 2018
circ_0001721	Human	Facilitates the progression of osteosarcoma malignancy	miR-569, miR-599	Li L. et al., 2018
circ_0016760	Human	Involves in non-small cell lung cancer by regulating GAGE1 expression	miR-1287	Li Y. et al., 2018
The circ_0007534	Human	Pancreatic ductal adenocarcinoma	miR-625, miR-892b	Hao et al., 2019
circUVRAG	Human Mouse	Bladder cancer	miR-223	Yang et al., 2019
circ_0058063	Human	Bladder cancer	miR145-5p	Sun et al., 2019
circ-U2AF1	Human	Glioma malignancy	miR-7-5p	Li et al., 2019
circ-UBAP2	Human	Triple-negative breast cancer	miR-661	Wang et al., 2018
**Tumor Suppressor Circrnas**				
circ-ITCH	Human Mouse	Bladder cancer inhibition by up regulation of p21 and PTEN	miR-17, miR-224	Yang C. et al., 2018
Cdr1/ciRS-7	Human Mouse	Bladder cancer	miR-135a	Li P. et al., 2018
circMTO1	Human	Hepatocellular carcinoma	miR-9	Han et al., 2017
circC3P1	Human	Hepatocellular carcinoma	miR-4641	Zhong et al., 2018
circ_0026344	Human	Colorectal cancer	miR-21, miR-31	Yuan et al., 2018
**Circrnas and Kidney**				
circHLA-C	human	Lupus glomerulonephritis	miR-150	Luan et al., 2018
ciRs-126	Human	Acute kidney injury	miR-126-5p	Kolling et al., 2018
circRNA_1297 circRNA 0528	Rat	kidney calculi	circRNA_162 rno-miR_672-5p	Lu et al., 2016; Cao et al., 2018
**Circrnas and Cardiovascular Disease**				
circANRIL	Human	Atherosclerosis and modulates PES1	Not identified	Holdt et al., 2016
circ_010567	Mouse	Diabetic mice myocardium and cardiac fibroblasts	miR-141	Zhou and Yu, 2017
circRNA MFACR	Mouse	Myocardial infarction	miR-652-3p	Wang K. et al., 2017
The autophagy-related circular RNA (ACR)	Mouse	Heart ischemia/reperfusion injury. Represses Pink1 and FAM65B	Not identified	Zhou L. Y. et al., 2018
rno-circ_0056717	Rat	Balloon-injured carotid artery	Not identified	Xu et al., 2018
hsa_circ_0037911	Human	Essential hypertension marker	Not identified	Bao et al., 2018
circRNA_000203, and circRNA_010567	Mouse	Overexpressed in the myocardium of diabetic mice and in cardiac fibroblasts	miR-26b-5p, miR-141	Tang et al., 2017; Zhou and Yu, 2017
**Circrnas in Other Diseases**				
circANKRD36	Human	Type 2 diabetes mellitus	hsa-miR-498, hsa-miR501-5p, and hsa-miR-3614-3p	Fang et al., 2018
circ_0005015	Human	Diabetic retinopathy	miR-519d-3p	Zhang et al., 2017
circRNA_Atp9b	Mouse	Mouse osteoarthritis	miR-138-5p	Zhou et al., 2018a
hsa_circRNA_102682, has_CircRNA_104820, has_circRNA_100782	Human	Up regulated in pre-eclampsia	Not identified	Qian et al., 2016
circ_101222	Human	Pre-eclampsia predictor	Not identified	Zhang et al., 2016
hsa_circ_0000650	Human	Hepatitis B	miR-6873-3p	Zhou T. C. et al., 2018
circ-Foxo3	Human Mouse	Cellular senescence by interacting with CDK2 and p21	Not identify	Du et al., 2016, 2017a
hsa_circ_0005836, hsa_circ_0009128	Human	Active pulmonary tuberculosis markers	Not identified	Zhuang et al., 2017

### circRNAs as Regulator of miRNA Function

There is a breadth of evidence to support the sponging effect (as competing endogenous RNA) regarding miRNAs of circRNAs in a tissue or organ specific manner. The following section describes several selected examples that demonstrate the specific sponging role of circRNAs in various tissues. The circRNA sponging function was elegantly demonstrated by Memczak et al. in their seminal study. They described the sponging function of a circRNA CDR1as or ciRS-7. This circRNA was shown to harbor 74 miR-7 seed matches suggesting a strong interaction between this circRNA and its miRNA target. The interplay between CDR1as and miR-7 was demonstrated to be specific to neuronal tissue in adult and embryonic mouse brain. CDR1as overexpression and the resulting inhibtion of miR-7 by morpholinos in zebrafish resulted in reduction of midbrain size (Memczak et al., 2013). Similarly, the testis specific circRNA *sex-determining region Y* (*Sry*) was shown to particularly sponge miR-138 and regulate its function in an *in vitro* study using HEK293 cellls (Hansen et al., 2013). The circRNA derived from exon 2 of the homeodomain-interacting protein kinase 3 (*HIPK3)* gene, circHIPK3 is thought to have the potential to sponge up to 9 different miRNAs with an estimation of 18 binding sites. There was a significant inhibition of cell growth upon circHIPK3 silencing in the human hepatocyte cell line HuH-7. Moreover the authors demonstrated direct binding to and sponging of miR-124 thus regulating its activity (Zheng et al., 2016). The expression of type 1 collagen is regulated by circCOL3A1-859267 in human dermal fibroblasts. Although this circRNA contains putative binding sites to several miRNA (e.g., miR29a-c, miR-767, and miR-133a), only miR29c was identified to be a target of circCOL3A1-859267. Overexpression of miR29c decreased type 1 collagen expression. However, this effect was abrogated by the introduction of circCOL3A1-859267 using a lentivirus expression system (Peng et al., 2018). In skeletal muscle development the circular RNA zinc finger protein 609 (circZfp609) suppressed the myogenic differentiation in mouse myoblast cell line C2C12 by sponging up miR-194-5p that regulates the expression of *BCL2-associated transcription factor (BCLAF1)* (Wang et al., 2019). Lastly, the suppression of circRNA.2837 expression may protect neurons from neurological damage in a rat sciatic nerve injury model since circRNA.2837 acts as a sponge to members of the miR-34 family (Zhou et al., 2018b).

### circRNAs and Cancer

Since the identification of circRNA sponging of miRNA that are involved in cancer pathogenesis, a multitude of studies have been forwarded regarding the association of circRNAs with various human cancers (Kristensen et al., 2018). Given the biomarker potential of circRNAs as well as the their proposed future use as therapeutic agents, it is imperative to improve our understanding how circRNAs and their miRNA targets are regulated in the development and progression of cancer. Therefore, in this review we will highlight the current findings that implicate certain circRNAs in cancer development and growth, in contrast to other circRNAs that act as tumor suppressors.

#### Oncogenic circRNAs

Several studies have outlined the sponging activity of certain circRNAs with regard to miRNAs, which are critical regulators of oncogenes in tumor development. For instance, overexpression of hsa_circ_0078710 increased cellular proliferation and migration, tumor growth and migration in human hepatocellular carcinoma (HCC) cell lines HepG2 and SMMC-7721 by sponging miR-31, increasing the expression levels of histone deacetylase 2 (HDAC2) and cyclin-dependent kinase 2 (CDK2) (Xie et al., 2018). In another HCC study the downregulation of the circRNA *filamin-binding LIM protein 1* (circFBLIM1) reduced cellular proliferation and invasion, and concomitantly induced apoptosis in human HCC cell lines HepG2, 7402, and 97H. CircFBLIM1 acts as a competitive endogenous RNA to filamin-binding LIM protein 1 (FBLIM1) and by binding to miR-346 which targets FBLIM1 mRNA (Bai et al., 2018). Circ_0001721 sponges miR-569 and miR-599 and facilitates the progression of osteosarcoma malignancy in humans. Therefore, the expression of circ_0001721 can be used as an unfavorable predictor of disease progression in osteosarcoma patients (Li L. et al., 2018). The oncogenic effect of circ_0016760 is attributed to the sponging activity of miR-1287 and the increased expression of cancer/testis antigen family 4 member 1 (GAGE1) in non-small cell lung cancer human cell lines A549 and H1299 (Li Y. et al., 2018). Circ_0007534 is found upregulated in patients with pancreatic ductal adenocarcinoma (PDAC). Overexpression of this circRNA was associated with advanced tumor stage and invasion of the lymph node. Further analysis revealed that overexpression of circ_0007534 in the human PDAC cell line SW1990 led to increased migration, proliferation, and invasion. Circ_0007534 mediates its oncogenic effect by sponging miR-625, and miR-892b (Hao et al., 2019). The downregulation of the circRNA *UV radiation resistance-associated gene* (circUVRAG) expression suppressed proliferation and metastasis of bladder cancer *in vitro* using the human bladder cancer cell line UM-UC-3 and *in vivo* using Balb/c nude mice injected with lentivirally stabilized circUVRAG UM-UC-3 into the right flank. The effects of circUVRAG are attributed to a mechanism that involves miR-223 sponging and downregulation of fibroblast growth factor receptor 2 (FGFR2) (Yang et al., 2019). Similarly, the oncogenic effect of circ_0058063 was demonstrated in another bladder cancer study. The suppression of circ_0058063 reduced cellular proliferation and migration and induced apoptosis in the human bladder cancer cell lines T24 and J82. The sponging of miR145-5p, which regulates cyclin-dependent kinase (CDK6), is a key element of the function of circ_0058063 in the development and progression of bladder cancer (Sun et al., 2019). In a human glioma malignancy study the circRNA U2 auxiliary factor 35 kDa subunit (circ-U2AF1) was found upregulated along with *neuro-oncological ventral antigen 2 (NOVA2)*. Suppression of circ-U2AF1 resulted in decreased expression of *NOVA2* and an increase in expression of miR-7-5p both *in vitro* and *in vivo*. As a result, silencing of circ-U2AF1 decreased cellular proliferation, migration and invasion, and increased cell death by apoptosis in the human glioma cell lines U87MG, and U251. Luciferase assay demonstrated that miR-7-5p is a direct target of circ-U2AF1 (Li et al., 2019). In triple-negative breast cancer (TNBC) circ-UBAP2 was found upregulated and associated with larger tumor size. The microRNA miR-661, which regulates the expression of the *metastasis-associated gene (MTA1)*, was found to be a direct target of circ-UBAP2. Suppression of circ-UBAP2 induced apoptosis and decreased proliferation and migration of the human TNBC cell lines BT-20 and MDA-MB-231 (Wang et al., 2018).

#### Tumor Suppressor circRNAs

Many studies have shown the remarkable potential that circRNAs may have in the future as an anti-cancer treatment option. The anti-tumor activity of the circRNA *itchy E3 ubiquitin protein ligase* (circ-ITCH) was demonstrated in human bladder cancer tissue and in human bladder cancer cell lines EJ and T24. The overexpression of circ-ITCH inhibited cellular migration, proliferation, and metastasis *in vitro* and also suppressed cancer formation of xenografts in Balb/c nude mice *in vivo*. The mechanism of circ-ITCH function is demonstrated by the up-regulation of the tumor suppressor genes p21 and phosphatase and tensin homolog (PTEN) expression due to the sponging effect of circ-ITCH to both miR-17 and miR-224 (Yang C. et al., 2018). Similarly, in another bladder cancer study the circRNA Cdr1 (also known as ciRS-7) is downregulated in cancer cells as compared to normal adjacent tissue. Overexpression of Cdr1 reduced cellular proliferation, migration, and invasion in the human bladder cancer cell lines EJ and T24 *in vitro*, and retarded tumor growth in Balb/c nude mice *in vivo*. The observed anti-oncogenic effect of Cdr1 is attributed to the sponging function of miR-135a (Li P. et al., 2018). Han et al. performed a global circRNA expression analysis using 289 human samples of HCC and paired adjacent liver tissues to identify dysregulated circRNA expression in HCC samples. Here, the circRNA *mitochondrial tRNA translation optimization 1* (circMTO1) was found to be significantly downregulated. Further analysis showed that suppression of circMTO1 led to increased proliferation and invasion of the human HCC cell lines HepG2 and SMMC-7721. CircMTO1 exerts its anti-tumor activity by sponging oncogenic miR-9 and thus preserving the expression of p21 (Han et al., 2017). The expression level of the circRNA complement component 3 precursor pseudogene (circC3P1) is significantly downregulated in HCC and the expression level of this circRNA is inversely correlated with tumor size and vascular invasion. In contrast, overexpression of circC3P1 decreased cellular migration, proliferation and invasion in human HCC Hep3B and MHCC97-L via a mechanism that involves sponging of miR-4641 and increased expression of phosphoenolpyruvate carboxykinase 1 (PCK1) (Zhong et al., 2018). Yuan et al. screened 32 pairs of human colorectal cancer (CRC) tissues and adjancent normal tissues and identified circ_0026344 to be downregulated in CRC tissues, whereas miR-21 and miR-31 expression levels were elevated. The ectopic expression of circ_0026344 induced apoptosis and reduced tumor growth and invasion in human CRC cell lines SW480 and HT29 via a mechanism that involves miR-21 and miR-31 sponging (Yuan et al., 2018).

### circRNAs and Kidney

The function of circRNAs in kidney homeostasis and disease pathogenesis remains largely unexplored. The few available studies, however, suggest that circRNAs may be used as promising biomarkers in blood (Memczak et al., 2015) and urine (Vo et al., 2019) of patients and may serve as therapeutic targets in kidney disease. Luan et al. performed a circRNA profiling in 6 kidney biopsies of patients with lupus nephritis (LN) compared to 5 healthy kidney tissues. Consequently, 171 circRNAs with at least twofold differential expression were identified. Interestingly, the expression of circHLA-C correlated positively with renal activity index, serum creatinine, proteinuria, and crescentic glomureli. The expression of circHLA-C in patients with LN was increased 2.72-fold compared to controls. This increased expression coincided with a 66% reduction in expression of miR-150 in LN patients. The authors argued that the sponging of miR-150 could be a factor in the pathogenesis of LN (Luan et al., 2018). In a global circRNA analysis of RNA extracted from blood of patients with acute kidney injury (AKI) three novel circRNAs were upregulated in the blood of AKI patients as compared to control. Most notably, the circRNA *circular RNA sponge of miR-126* (ciRs-126) was found to be an independent predictor of 28 days survival of patients with acute kidney injury (AKI). In this study miR-126-5p was downregulated in AKI patients and in hypoxic endothelial cells. This effect is attributed to the potential sponging function of ciRs-126 of its target miR-126-5p (Kolling et al., 2018). Wang et al studied the expression of circRNAs in uremia due to glomerulonephritis. They screened a total of 20 individuals, 10 healthy controls as well as 10 patients without systemic lupus erythematosus, for the presence of circRNAs in plasma and peripheral mononuclear cells (PBMCs). The research group identified 385-upregulated and 325 downregulated circRNAs in plasma. In addition, 670 upregulated and 298 downregulated circRNAs were found in PBMCs. The differentially expressed circRNAs relate to various cellular functions including signal transduction, migration, cell differentiation, immune responses and other functions. For example, the regulated circRNAs target genes include ring finger protein 41 (RNF41), ST6 beta-galactoside alpha-2,6-sialtransferase (ST6GAL1), WD repeat-containing protein 37 (WDR37), notch homolog 1, translocation-associated (Notch), protein phosphatase 2, regulatory subunit B′, alpha subunit (PPP2R5A), adaptor related protein complex 4 subunit Mu 1 (AP4M1), and presenilin 1 (PSEN1). The authors suggest that this study could potentially provide diagnostic biomarkers for the diagnosis and prognosis of patients with uremia (Wang X. et al., 2017). The expression profiles of circRNAs in normal and fetal human tissues using RNA-seq identified 474 circRNAs with adult kidney specific expression and 7962 circRNAs expressed in the human fetal kidney. These results suggest that circRNAs function in a tissue specific manner (Xu et al., 2017). In a rat model of ethylene glycol-induced kidney calculi the expression profiles of mRNA, circRNAs linear lncRNAs were determined. In this animal model 145 circRNAs were deregulated in rat kidney with urolithiasis. Interestingly, circRNA_1297 was associated with rno-miR-138-5p, but circRNA_0528, and circRNA_162 were associated with rno-miR_672-5p. The two microRNAs have been demonstrated to play a role in the development of hypercalciuria urolithiasis (Lu et al., 2016; Cao et al., 2018).

### circRNAs and Cardiovascular Diseases

The role of circRNA in the pathogenesis of cardiovascular diseases is a heavily reviewed topic (Fan et al., 2017; Althesha et al., 2018; Carrara et al., 2018; Holdt et al., 2018a,b; Stȩpień et al., 2018; Zhou M. Y. et al., 2018). Therefore a summary of the major findings will be described here. The atherosclerosis protective circRNA antisense non-coding RNA in the INK4 locus (circANRIL) modulates the function of *pescadillo homologue 1 (PES1)*, an important 60S-preribosomal assembly factor, and protects against atherogenesis by modulating ribosomal RNA (rRNA) maturation in human smooth muscle cells and macrophages, thereby inducing apoptosis and inhibiting cellular proliferation (Holdt et al., 2016). In a mouse study, the expression of circ_010567 was increased in diabetic mice myocardium and cardiac fibroblasts via a mechanism that involved miR-141 sponging and upregulation of transforming growth factor beta 1 (TGFβ1) expression. The suppression of circ_010567 expression reduced fibrotic protein expression such as Collagen 1, Collagen 3 and alpha smooth muscle actin (αSMA) in mouse cardiac fibroblasts, (Zhou and Yu, 2017). The circRNA *MFACR (mitochondrial fission and apoptosis-related circular RNA)* targets miR-652-3p specifically that leads to mitochondrial fission inhibition and a reduction of cardiomyocyte apoptosis by decreasing the expression of *mitochondrial protein 18 (MTP18)*. In a mouse model of myocardial infarction (MI), suppression of *MFACR* in cardiomyocytes decreased mitochondrial fission and notably myocardial infarction (Wang K. et al., 2017). The function of circRNAs in the regulation of autophagy in cardiovascular diseases was demonstrated by Zhou et al. The *Autophagy-related circular RNA (ACR)* has a protective role in the heart against ischemia/reperfusion injury in a mouse model of myocardial infarction by repressing autophagy through a process that involves PTEN induced putative kinase 1 (Pink1) activation, family with sequence similarity 65, member B (FAM65B) phosphorylation, and the blocking of Pink1 promoter DNA methylation mediated by DNA methyltransferase 3 beta (Dnmt3B) (Zhou L. Y. et al., 2018). In a rat model of balloon-injury of the carotid artery, the diaphanous related formin 3 (Diaph3) derived *circRNA rno-circ_0056717 (circDiaph3)* was identified in a microarray and the suppression of this circRNA increased insulin-like growth factor 1 receptor (Igf1r) expression and stimulated vascular smooth muscle cell proliferation and migration (Xu et al., 2018). In an essential hypertension (EH) study that included 200 patients, the circRNA *hsa_circ_0037911* was significantly overexpressed in EH patients and the authors suggest that this circRNA could be an effective marker of EH (Bao et al., 2018). The circRNAs *circRNA_000203*, and *circRNA_010567* are overexpressed in the myocardium of diabetic mice and in cardiac fibroblasts in a cardiac fibrosis mouse model related to Angiotensin II (Ang II). Overexpression of circRNA_000203 in mouse cardiac fibroblasts resulted in a promotion of the expression of collagen-1-α2 (col1a2), collagen-3-α1 (col3a1) and αSMA via a mechanism of sponging miR-26b-5p. Additionally, the downregulation of circRNA_010567 in cardiac fibroblasts resulted in suppression of TGFb1 expression, which is a target of miR-141, and the decreased expression of fibrosis related genes (Tang et al., 2017; Zhou and Yu, 2017).

### circRNAs in Other Diseases

The role that circRNAs play in disease protection or development extends to several other diseases that have been already reported. For instance, the circRNA derived from the gene ankyrin repeat domain-containing protein 36A (circANKRD36) was overexpressed in peripheral leucocytes of patients with type 2 diabetes mellitus (T2DM). The expression of circANKRD36 positively correlated with higher glucose and glycosylated hemoglobin levels. In addition, the expression levels of interleukin-6 (IL-6) and tumor necrosis factor alpha (TNF-alpha) were significantly increased in the peripheral blood of patients with T2DM. The authors suggest an involvement of circANKRD36 in the increased inflammatory response in T2DM patients through a mechanism that involves the regulation of miRNAs, hsa-miR-498, hsa-miR501-5p, and hsa-miR-3614-3p (Fang et al., 2018). In diabetic retinopathy patients the expression of circ_0005015 was elevated in the fibrovascular membranes, the plasma and the vitreous sample, which resulted in enhanced endothelial angiogenic responses via sponging miR-519d-3p and increased expression of matrix metallopeptidase 2 (MMP-2), signal transducer and activator of transcription 3 (STAT3), and X-linked inhibitor of apoptosis (XIAP) (Zhang et al., 2017). The function of circRNA derived from the gene ATPase phospholipid transporting 9B (circRNA_Atp9b) was examined in a mouse osteoarthritis chondrocyte cell model. CircRNA_Atp9b expression level was elevated in mouse chondrocytes in response to interleukin-1β (Il-1β) treatment. Suppression of CircRNA_Atp9b expression resulted in decreased matrix metallopeptidase 13 (MMP13), interleukin-6 (IL-6), and cyclooxygenase 2 (COX-2) expression while type II collagen was increased. The regulatory function of circRNA_Atp9b in the progression of osteoarthritis is attributed to the sponging of miR-138-5p in mouse chondrocytes (Zhou et al., 2018a). In a study involving placental tissues to identify circRNA dysregulation in pre-eclampsia by microarray, 143 circRNAs were induced, while 158 were repressed as compared to controls. The top three circRNAs that were significantly upregulated were hsa_circRNA_102682, hsa_circRNA_104820 and hsa_circRNA_100782 (Qian et al., 2016). However the function of these circRNAs in the pathogenesis of pre-eclampsia has not been elucidated to date. Similarly, circ_101222 could be used as pre-eclampsia predictor in combination with the expression levels of endoglin in plasma (Zhang et al., 2016). RNA sequencing involving liver biopsies of chronic hepatitis B virus infection revealed a total of 99 dysregulated circRNAs as compared to control. Computational and regression analyses showed a positive correlation between hsa_circ_0000650 and TGF-beta-2 with a possible interaction between miR-6873-3p and TGF-beta-2. Taken together these results suggest an involvement of circRNAs in the pathogenesis of hepatitis B virus infection and the progression of liver disease (Zhou T. C. et al., 2018). New evidence implicates circRNAs in aging and cellular senescence. Results of RNA-seq from cortex and hippocampus of young as compared to aged mice showed a significant accumulation of circRNAs in brain tissues (Gruner et al., 2016). The circRNA derived from forkhead box O3 (Foxo3) gene termed circ-Foxo3 is implicated in cellular senescence as overexpression of this circRNA promoted senescence while silencing circ-Foxo3 suppressed cellular senescence. Circ-Foxo3 exerts its effects by binding to *cyclin-dependent kinase 2 (CDK2)* and the cell cycle inhibitor p21 (Du et al., 2016, 2017a). Two circRNAs hsa_circ_0005836 and hsa_circ_0009128 were identified using high-throughput sequencing to be downregulated in PBMCs in patients with active pulmonary tuberculosis compared to controls. However, the authors suggest that only hsa_circ_0005836 could be used potentially as a novel tuberculosis biomarker (Zhuang et al., 2017).

## Conclusion

The elucidation of the function of circRNAs is an emerging field of science with a tremendous potential after previously being dismissed as RNA artifacts. They are ubiquitously expressed and thousands of members have already been identified. This fact only expands their potential to possibly enhance our knowledge to understand the difference between health and disease. Owing to their structure stability and their presence in exosomes circRNAs may also exert their function in an autocrine, paracrine and possible endocrine fashion. In addition, the fact that circRNAs are widely distributed in the cellular compartment as well as in the extracellular space in various body fluids affords them to become ideal candidates as reliable biomarkers for various human diseases. As in any emerging field caution is advised in interpreting new findings. To date studies are merely descriptive in nature. More in depth analysis about the function and regulation of identified circRNAs is needed to understand their mechanism of action. These RNA molecules hold great promise as biomarkers of disease because of their high resistance to exonuclease activity and might even be more highly expressed than their linear counterparts. Especially their putative role as miRNA sponges makes them interesting targets for future investigations. In the future circRNA-based therapies might be introduced. The concept of establishing circRNAs as therapeutics in the future relies on the stability and expression in distinct organs and their specificity for a certain disease. The more specific circRNAs are to a specific organ or disease, the less likely it is to encounter off-target effects, while therapeutically modulating a specific circRNA in a disease setting. As is known for microRNA the likelihood of ensuing significant and undesired off-target effects greatly limits the use of these transcripts as therapeutics. The elucidation of the role of specific circRNAs is the prerequisite for a targeted RNA–based therapy for a specific disease. Tissue/cell specificity might be achieved by coupling ncRNAs to tissue-specific antibodies and/or peptides, thereby reducing off-target effects.

## Author Contributions

GH and JL contributed equally to the review manuscript. Both authors approved the final manuscript for submission and publication.

## Conflict of Interest Statement

The authors declare that the research was conducted in the absence of any commercial or financial relationships that could be construed as a potential conflict of interest.
